# Probing the Association between Early Evolutionary Markers and Schizophrenia

**DOI:** 10.1371/journal.pone.0169227

**Published:** 2017-01-12

**Authors:** Saurabh Srinivasan, Francesco Bettella, Sahar Hassani, Yunpeng Wang, Aree Witoelar, Andrew J. Schork, Wesley K. Thompson, David A. Collier, Rahul S. Desikan, Ingrid Melle, Anders M. Dale, Srdjan Djurovic, Ole A. Andreassen

**Affiliations:** 1 NORMENT, KG Jebsen Centre for Psychosis Research, Institute of Clinical Medicine, University of Oslo, Oslo, Norway; 2 Division of Mental Health and Addiction, Oslo University Hospital, Oslo, Norway; 3 Multimodal Imaging Laboratory, University of California at San Diego, La Jolla, CA, United States of America; 4 Cognitive Sciences Graduate Program, University of California, San Diego, La Jolla, CA, United States of America; 5 Center for Human Development, University of California at San Diego, La Jolla, CA, United States of America; 6 Institute of Biological Psychiatry, Mental Health Center St. Hans, Mental Health Services Copenhagen, Roskilde, Denmark; 7 The Lundbeck Foundation Initiative for Integrative Psychiatric Research, iPSYCH, Department of Clinical Medicine, University of Copenhagen, Copenhagen, Denmark; 8 Eli Lilly & Co, Erl Wood Manor, Windlesham, Surrey, United Kingdom; 9 Neuroradiology Section, Department of Radiology and Biomedical Imaging, University of California, San Francisco, CA, United States of America; 10 Department of Neuroscience, University of California at San Diego, La Jolla, CA, United States of America; 11 Neuroradiology Section, Department of Radiology and Biomedical Imaging, University of California at San Francisco, San Francisco, CA, United States of America; 12 Department of Medical Genetics, Oslo University Hospital, Oslo, Norway; 13 NORMENT, KG Jebsen Centre for Psychosis Research, Department of Clinical Science, University of Bergen, Bergen, Norway; SPAIN

## Abstract

Schizophrenia is suggested to be a by-product of the evolution in humans, a compromise for our language, creative thinking and cognitive abilities, and thus, essentially, a human disorder. The time of its origin during the course of human evolution remains unclear. Here we investigate several markers of early human evolution and their relationship to the genetic risk of schizophrenia. We tested the schizophrenia evolutionary hypothesis by analyzing genome-wide association studies of schizophrenia and other human phenotypes in a statistical framework suited for polygenic architectures. We analyzed evolutionary proxy measures: human accelerated regions, segmental duplications, and ohnologs, representing various time periods of human evolution for overlap with the human genomic loci associated with schizophrenia. Polygenic enrichment plots suggest a higher prevalence of schizophrenia associations in human accelerated regions, segmental duplications and ohnologs. However, the enrichment is mostly accounted for by linkage disequilibrium, especially with functional elements like introns and untranslated regions. Our results did not provide clear evidence that markers of early human evolution are more likely associated with schizophrenia. While SNPs associated with schizophrenia are enriched in HAR, Ohno and SD regions, the enrichment seems to be mediated by affiliation to known genomic enrichment categories. Taken together with previous results, these findings suggest that schizophrenia risk may have mainly developed more recently in human evolution.

## Introduction

Schizophrenia has affected humans throughout history; it has heritability between 60–80% [1] and a global prevalence of around 1% [2, 3]. It is characterized by hallucinations and delusions, often involving language and thought disorders, and higher order cognitive dysfunction [4]. Hence, schizophrenia has been suggested to represent, in part, a by-product of adaptive changes during the hominization process [3, 5]. Archaeological and paleontological findings may provide us with evidence of the cultural and anatomical changes in skeletal structure, however, mental and psychiatric changes are hard to trace.

Humans differ from their ancestors on a range of skills including language, creativity, metacognition, executive function, and cooperation. [6, 7] These are important characteristics that involved genetic changes leading to functional advantages, with cultural and societal effects. These changes which set us apart from our ancestors could have also made us vulnerable to psychiatric disorders like schizophrenia, plausibly a human-specific disease. [5, 8]

It is still unknown at which stage of human evolution the risk factors for schizophrenia emerged. We have previously found evidence that genetic risk appeared during the divergence of modern *Homo sapiens* from *Homo Neanderthalensis*. [9] As every stage of evolution was driven by genetic changes, it is possible that gene variants associated with schizophrenia emerged even earlier, for example, when new world and old world monkeys split into different branches of the evolutionary tree [10], or even as early as when vertebrates appeared on earth. [11]Building on the recent progress in genome-wide association studies (GWAS) [12], we now have the opportunity to investigate the origin of psychosis further back in the evolution. The human genome consists of evolutionarily new and ancient regions. By investigating single nucleotide polymorphisms (SNPs) association with schizophrenia in these regions, it is possible to roughly estimate when the risk potential appeared during evolution.

Here, we investigated schizophrenia SNPs located in human accelerated regions, segmental duplications and ohnologs to determine to what extent SNPs tagging these regions, which are proxies for various periods in early evolutionary history, are associated with schizophrenia or other human traits and diseases. These represent early evolutionary markers older than 200,000 years when modern humans are regarded to have first appeared on earth. [13] Human accelerated regions (HAR) are DNA sequences that experienced rapid changes after the divergence of humans (including our hominin ancestors) from chimpanzees after remaining constant throughout primate evolution. [14] While most HARs are exclusively non-coding sequences, research suggests that many HARs are developmental gene regulatory elements and RNA genes, most of which evolved their uniquely human mutations through positive selection before divergence of archaic hominins and diversification of modern humans [15]. These regions harbor genes that have been shown to play important roles in neurocognitive development [14, 16]. It is known that various genomic regions show differential enrichment, [17] i.e. that certain genomic regions are more likely to harbor gene variants associated with human traits and diseases. Further, an enrichment of schizophrenia risk loci could be due to a general effect of brain related genes, [12] not necessarily of those implicated in evolution. Moreover, according to recent GWAS, gene variants at the major histocompatibility complex (MHC) region on human chromosome 6p22.1 are implicated in schizophrenia [12, 18, 19] and play a role in evolution. [20] Thus, it is important to disentangle the effect of these mediating factors from the effect of the evolutionary proxies.

Segmental duplications (SD), also known as low copy repeats, are a known source of genetic instability and evolution [10]. Most duplication in the hominid branch can be traced to events 35–40 million years ago [10], marking an early stage of hominid/primate evolution. Increased duplications are seen in regions that distinguish the hominid branch from other primates. Eichler et al. [21] found that the primate ancestral branch leading to human and African great apes showed the most significant increase in duplication activity both in terms of base pairs and in terms of events. In light of the importance of SDs in contributing to copy-number changes associated with neurocognitive disease [22], it is suggested that this apparent acceleration had a profound impact on the reproductive success, adaptability and evolution of ancestral hominid populations [21].

Ohnologs (Ohno) are genes retained after whole genome duplication events, unlike segmental duplications which are duplications of smaller chunks of the genome. They are often over represented in copy number variations (CNVs) that cause complex neurodevelopmental disorders like schizophrenia, autism spectrum disorders, neurodevelopmental delay, intellectual disability and epilepsy [15, 23]. Whole genome duplications events are said to have occurred early in the vertebrate lineage around 500 million years ago [24] and the human genome contains many more duplications than would be expected by chance. These are evolutionarily important since gene duplication and divergence are the primary source of new genes in eukaryotes [25, 26].

We used a statistical framework suited for polygenic architectures [17, 27]. These methods have been applied, with success, to GWAS of complex human phenotypes to probe the overlap between phenotypes association and Neanderthal selective sweep [9]. Here, we use them to investigate if SNPs in regions of early human evolution are enriched of association with schizophrenia, while controlling for potential confounders.

## Methods and Materials

### Samples

We obtained summary statistics for single nucleotide polymorphisms (SNPs) from genome-wide association studies (GWAS) of schizophrenia (conducted by the Psychiatric Genomics Consortium (PGC)) [12] and other phenotypes representing a selection of morphological, cardiovascular, immunological and psychiatric phenotypes. They include anthropometric measures (body mass index (BMI) [28], height) [29], a cardiovascular disease risk factor (triglycerides (TG)) [30], an immune-mediated disease (Crohn’s disease (CD)) [31] a neurological disorder (Alzheimer’s disease (AD) [32] as well as another psychiatric disorder (bipolar disorder (BD)) [33] (**S1 Table**). In order to make the data comparable all summary statistics were aligned to a set of about 2.5 million variants.

### Evolutionary proxies

We computed LD weighted HAR, SD and Ohno regional affiliation scores following the procedure detailed below.

#### Human accelerated region (HAR) score

The HAR score indicates a SNP’s affiliation to HAR. All SNPs were first assigned a raw HAR indicator value of 1 or 0 according to whether they fell inside or outside any HAR, and subsequently an LD-weighted score. The list of HAR was obtained from http://www.broadinstitute.org/scientific-community/science/projects/mammals-models/29-mammals-project-supplementary-info.

#### Human segmental duplication (SD) score

The human SD score indicates a SNP’s affiliation to SD regions in humans. All SNPs were first assigned a raw score of 1 or 0 and subsequently an LD-weighted score. The lists of segmental duplications were downloaded from the SD database at http://humanparalogy.gs.washington.edu/.

#### Ohnolog (Ohno) score

This score indicates a SNP’s affiliation to Ohno regions. All SNPs were first assigned a raw score of 1 or 0 and subsequently an LD-weighted score. The lists of Ohno regions was obtained from McLysaght et al. [25].

### Confounding/mediating factors

We investigated if the following factors could affect the evolutionary enrichment of schizophrenia associations.

#### Brain genes

We used the NCBI resource (http://www.ncbi.nlm.nih.gov/gene) to select all genes with any relation to the brain. We identified a total of 2494 genes by filtering specifically for genes in *Homo sapiens* matching the query phrase “human brain”. All 1000 Genomes Project SNPs in these genes were assigned a “Brain” value of 1, the rest were assigned a”Brain” value of 0. All SNPs were subsequently assigned LD–weighted “Brain” scores.

#### Major histocompatibility complex (MHC)

The MHC has been implicated in schizophrenia as well as a number of other phenotypes, particularly immune-mediated diseases. The evolution of MHC itself may have involved SD and other large scale genetic variations [34]. It is therefore reasonable to expect that SNPs in these regions might be confounding some of the evolutionary enrichment results. To test for the effect of MHC, we removed the SNPs in the MHC region (chromosome 6 region between genomic positions 25652429 and 33421466 in the hg19 assembly) and repeated the analyses.

#### Annotation of genomic regions (LD based)

The SNPs that fall within certain regions of interest may capture only a limited portion of the association signal actually ascribable to that region. We used an LD-weighted scoring algorithm [17] in order to identify SNPs that tag specific DNA regions even if they are not situated within them. For each SNP a pairwise correlation coefficient approximation to LD (*r*^2^) was extracted for all 1KGP SNPs within a 1,000,000 base pairs (1Mb). All *r*^2^ values < 0.2 were set to 0 and each SNP was assigned an *r*^2^ value of 1.0 with itself. LD-weighted region annotation scores for all DNA regions of interest were computed as the sum of LD *r*^2^ between the tag SNP and all 1KGP SNPs in those regions. Given SNP_*i*_, its LD-weighted region annotation score was computed as LDscore_*i*_ = *Σ*_*j*_ (*δ*_*j*_ *r*_*ij*_^2^), where *r*_*ij*_^2^ is the LD r-squared between SNP_*i*_ and SNP_*j*_ and *δ*_*j*_ takes values of 1 or 0 depending on whether the 1KGP SNP_*j*_ is within the region of interest or not.

LD scores were also assigned to exons, introns, 3’UTR and 5’UTR, and the total LD score (TotLD) was computed following the same procedure but extending the tagging region to the whole 1Mb window.

#### Intergenic correction

Intergenic SNPs are defined as having LD-weighted annotation scores for exon, intron, 3’UTR and 5’UTR equal to zero and being in LD with no SNPs in the 1KGP reference panel located within 100,000 base pairs of a protein coding gene, within a non-coding RNA, within a transcription factor binding site or within a miRNA binding site. Those singled out in this way are expected to form a collection of non-genic SNPs not belonging to any (annotated) functional elements within the genome (including through LD) and therefore represent a collection of likely null associations. Intergenic SNPs were used to estimate the inflation of GWAS summary statistics due to cryptic relatedness. We used intergenic SNPs because their relative depletion of associations (17) suggests they provide a set of reliably null SNPs that is less contaminated by polygenic effects. The inflation factor, λ_GC_, was estimated as the median squared z-score of independent sets of intergenic SNPs across one hundred LD-pruning iterations, divided by the expected median of a chi-square distribution with one degree of freedom.

### Analytical approach

We employed a genetic enrichment method recently developed to uncover more of the genetic architecture of complex traits [17, 35–38]. Specifically, we investigated the enrichment of associations concurrent with the evolutionary affiliations in a covariate-modulated statistical framework [37]. We investigated whether SNPs located in the evolutionarily salient regions (HAR, SD, Ohno) or tagging other SNPs therein, are more likely associated with schizophrenia or other phenotypes using GWAS data from existing non-censored summary statistics.

All statistical analyses were carried out with a covariate-modulated enrichment analysis package developed on R (www.r-project.org) and MATLAB (www.mathworks.se/products/matlab/) programming platforms.

#### Fold enrichment plots

To visually assess genetic enrichment, we used conditional fold enrichment plots [39]. For this purpose the covariate of interest, i.e. the region affiliation score, is used to subdivide SNPs into two strata. For LD-weighted annotation scores, the choice of a threshold score is somewhat arbitrary. We chose 1 since this is the score an isolated SNP within a salient region would have. It has been shown elsewhere [17] that the method is robust to the choice of threshold.

The enrichment plots were obtained by computing the empirical cumulative distribution of–log10(p)-values for SNP association with a given phenotype for all SNPs, and for the two dichotomous SNPs strata determined by the region affiliation score. Then each stratum’s fold enrichment was calculated as the ratio CDF_stratum_/CDF_all_ between the–log10(p) cumulative distribution for that stratum and the–log10(p) cumulative distribution for all SNPs. The nominal–log10(p) values are plotted on the x-axis, the fold enrichment in the y-axis. To assess polygenic effects below the standard GWAS significance threshold, we focused the fold enrichment plots on SNPs with nominal–log10(p) < 7.3 (corresponding to p > 5x10^-8^). Enrichment is present if the line corresponding to the SNPs of interest has a positive deflection from the horizontal line through 1.

#### Partial least squares regression (PLSR)

The fold enrichment plots give a visual impression of the different association propensities of SNPs affiliated to the evolutionarily salient regions. However, they do not give a quantitative measure of the eventual enrichment. One such measure is provided by squared association z-scores regression [9] which in addition allows controlling for covariates of no interest. Due to their nature, the effects of LD-weighted annotation scores can’t be estimated by standard linear regression.

PLSR is a supervised subspace regression method that maximizes covariance between two data blocks: the so called descriptor data set (X) and response data set (Y) [40]. An important aspect of PLSR is that the regression model is statistically stabilized for data sets with highly inter-correlated variables [41]. The predictive PLSR model may be written as follows:

$$Y=XB_{A}+F_{A}$$

Where X and Y are descriptor and response data matrices respectively (both data sets are mean-centered and scaled prior to data modelling), *B*_*A*_ are the regression coefficients for a model including *A* latent variables (LVs) and *F*_*A*_ is the residual matrix for the corresponding model.

For all of the PLSR models in this study, we chose the optimal number of LVs (*A*) as the number of LVs that explained more than 99% of the variance in the descriptor data set.

#### Statistical validation: Jackknife approximate t-tests of regression coefficients

In order to assess the contribution of the descriptor variables to the PLSR model we carried out approximate t-tests of regression coefficients based on jackknife variance estimates [42]. For this purpose, we ran 50-fold cross-validation on the SNP samples and re-calculated the regression coefficients in every cross-validation round. The LD-matrix was used for partitioning the SNP samples into the cross-validation subsets. We then calculated jackknife estimates for the standard deviations of the regression coefficients and, thereafter, t-statistics and approximate p-values indicating the significance of the association with the corresponding descriptor variables in the PLSR model.

#### Squared z-scores residuals versus TotLD stratified scatter plots

The PLSR results are better visualized by plotting descriptor and response variables directly. We residualized the squared z-scores in PLSR models deprived of TotLD, binned the latter and plotted the average squared z-scores for all bins against the corresponding TotLD bin centers. To control for the effect of the evolutionary measures we residualized the squared z-scores in a second series of PLSR models deprived of TotLD as well as the evolutionary measure of interest and again plotted the average squared z-scores for all bins against the corresponding TotLD bin centers.

## Results

**Fig 1:**
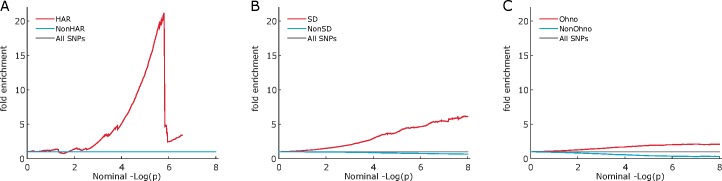
ABC: Enrichment plots for schizophrenia, stratifying SNPs according to their affiliation to human accelerated regions segmental duplications and ohnologs. Plot A shows all SNPs stratified by affiliation to human accelerated regions (HAR) and non (NonHAR). Plot B shows all SNPs stratified by affiliation to segmental duplications (SD) and non (NonSD). Plot C shows all SNPs stratified by affiliation to ohnologs (Ohno) and non (NonOhno). Some enrichment could be present in HAR but the drop at the leftmost end of the plot suggests low SNP count and consequent high error rate. A clearer enrichment is present in segmental duplications and a similar but weaker one in ohnologs. All annotations are LD-weighted.

We assessed the influence exerted on schizophrenia association propensity by affiliation to HAR, SD and Ohno. The fold enrichment plots (**Fig 1**) suggest enrichment of schizophrenia association among SNPs in HAR and SD regions, and to some extent, Ohno.

MHC has a known association with schizophrenia [18]. To assess the effect of immune-related genes, the analyses were repeated after exclusion of SNPs in the MHC region. The most significant reduction in fold enrichment occurred with SD but none was visible in HAR or Ohno **(S1 Fig)**.

**Fig 2:**
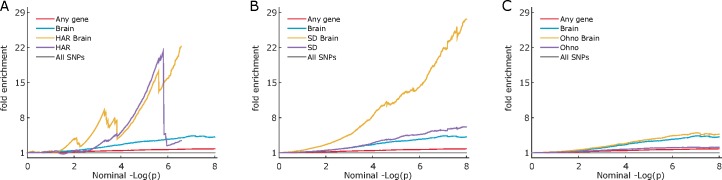
ABC: Enrichment plots showing schizophrenia association enrichment of brain genes with various regional affiliation scores. Enrichment plots for A) human accelerated regions (HAR), B) segmental duplications (SD) and C) ohnologs are shown for: SNPs annotated to genes with some relation to the brain, as established by an NCBI site search (“Brain”); SNPs affiliated to these regions of interest (HAR, SD or Ohno) and also annotated to genes with some relation to the brain (HAR Brain, SD Brain or Ohno Brain). In case of segmental duplications, the brain genes in the regions of interest (SD Brain) look more enriched (i.e. present a higher incidence of associations [lower p-values] with schizophrenia) compared to SD or just any Brain genes. In Ohnologs, the enrichment is way lower than in SD but Ohno Brain looks more enriched than other categories. HAR Brain and HAR show similar enrichment. All annotations are LD-weighted.

To test if the enrichment seen in schizophrenia genes was mediated by brain function, we investigated the fold enrichment of brain genes annotated to various combinations of regions of evolutionary interest (**Fig 2).** We observe a rather wider deflection from baseline for brain genes affiliated to HAR and SD, suggesting brain genes in these evolutionarily salient regions are more enriched compared to just any brain genes or just any SNPs in HAR, SD or Ohno.

We then ascertained the effect exerted on enrichment by other known factors, using PLSR. Regressing association squared z-scores in turn against HAR, SD and Ohno affiliation scores with exonic, intronic, 5'UTR, 3'UTR [17, 43], Brain and TotLD suggest that these co-factors explain nearly all of the enrichment (**Tables 1–3A**; HAR, non-centered standardized coefficients *β* = 0.002, p = 0.57; SD, *β* = -1.847, p = 6.00x10^-6^; Ohno, *β* = 0.496, p = 0.694).

**Table 1:** Human accelerated regions (HAR): Partial least square regression (PLSR) coefficients with multiple covariates.

**A**							
**Phenotypes**	**Intron**	**Exon**	**3UTR**	**5UTR**	**HAR**	**Brain**	**TotLD**
**AD**	2.187^*^	0.244^*^	0.317^*^	0.073	0.000	2.134	-0.003
**BD**	1.874	0.233	0.201	0.115	0.003	2.492	1.302^*^
**SCZ**	2.583^*^	**0.189**	**0.342**	**0.144**	**0.002**	**1.568**	**1.627**
**BMI**	2.196^*^	-0.130	0.057	0.030	0.005	1.984	0.996
**Height**	2.551^**^	0.153	0.228^**^	0.108^*^	0.006	-0.598	0.644^**^
**TG**	2.435^**^	0.336	0.581	0.157	0.004	-0.870	0.377
**CD**	2.594^*^	0.860	0.767	0.365	-0.006	2.444^*^	1.433^*^
**B**							
**Phenotypes**	**Intron**	**Exon**	**3UTR**	**5UTR**	**HAR**	**Brain**	**TotLD**
**AD**	2.182^*^	0.251^*^	0.325^*^	0.067	0.000	2.141	-0.003
**BD**	1.953^*^	0.116	0.083	0.080^*^	0.003	2.374	1.044
**SCZ**	2.551^**^	**-0.059**	**0.163**	**0.043**	**0.002**	**1.497**	**1.125**
**BMI**	2.227^*^	-0.131	0.065	0.030	0.005	1.948	1.006
**Height**	2.560^**^	0.097	0.202^**^	0.083^*^	0.004	-0.565	0.530^**^
**TG**	2.482^**^	0.191	0.444	0.104	-0.001	-0.752	0.185
**CD**	2.660^**^	0.608	0.583	0.264	-0.007	2.172^*^	1.070^*^
**C**							
**Phenotypes**	**Intron**	**Exon**	**3UTR**	**5UTR**	**HAR**	**Brain**	**TotLD**
**AD**	2.511	-0.254^*^	-0.173	0.198	0.001	0.640	-0.333
**BD**	-0.006	0.001^*^	0.001	0.000	0.000	0.000	0.004
**SCZ**	**-1.164**	**0.660**	**0.331**	**0.028**	**-0.004**	**-0.961**	**1.801**
**BMI**	-2.363	0.112	-0.089	0.049	0.009	0.477	0.608
**Height**	-2.198	0.171	-0.085	0.070^*^	0.019	-0.026	1.118
**TG**	-1.206	0.553	0.394	0.162	0.039	0.292	2.194
**CD**	-1.599	0.358	0.103	0.168^*^	0.018	1.583	1.094

* nominally significant

** significant after accounting for multiple testing

Phenotypes: psychiatric and neurological diseases (Alzheimer’s disease (AD), bipolar disorder (BD), schizophrenia (SCZ)), anthropometric measures (body mass index (BMI), height), cardiovascular risk factors (Triglycerides (TG)), and immune-mediated diseases (Crohn’s disease (CD)). **A**: regression on the complete set of SNPs, **B**: regression analysis after removing MHC SNPs and **C**: regression analysis keeping only MHC SNPs. The visual enrichment seen in schizophrenia (**Fig 1A**) is not supported by regression where no significant (p = 0.57) association is detected. The results remain non-significant after removing MHC SNPs or using only them.

**Table 2:** Segmental duplication (SD): Partial least square regression (PLSR) coefficients with multiple covariates.

**A**							
**Phenotypes**	**Intron**	**Exon**	**3UTR**	**5UTR**	**SD**	**Brain**	**TotLD**
**AD**	2.577^**^	0.953	1.221	0.275	1.771	2.108	-0.231
**BD**	1.049	0.348	0.301	0.172	-1.668	1.368	0.960^*^
**SCZ**	1.190^*^	**0.271**	**0.495**	**0.205**	-1.847^**^	**0.541**	1.006^*^
**BMI**	1.065^*^	-0.140	0.065	0.032	-1.843^*^	1.010	0.739^*^
**Height**	2.797^**^	0.997	1.478	0.725	1.127	-0.502	0.555
**TG**	0.206	1.420	2.470	0.665	-0.445	0.027	0.092
**CD**	0.024	2.253^*^	1.987^**^	0.962^*^	-0.022	0.045	0.006
**B**							
**Phenotypes**	**Intron**	**Exon**	**3UTR**	**5UTR**	**SD**	**Brain**	**TotLD**
**AD**	2.559^**^	0.971	1.226	0.252	1.807	2.088	-0.234
**BD**	1.120^*^	0.153	0.106	0.102	-1.620	1.423	0.829^*^
**SCZ**	1.277^**^	**-0.073**	**0.197**	**0.049**	-1.816^**^	**0.743**	0.824^*^
**BMI**	1.074	-0.142	0.074	0.032	-1.849^*^	0.986	0.740^*^
**Height**	2.257^**^	1.095	2.365	1.004	0.743	-0.446	0.358^**^
**TG**	1.198^*^	0.840	1.983	0.456	-1.173	-0.080	0.253
**CD**	0.781	1.978	1.879	0.855	-0.993	0.488	0.432
C							
**Phenotypes**	**Intron**	**Exon**	**3UTR**	**5UTR**	**SD**	**Brain**	**TotLD**
**AD**	1.785	-0.876	-0.631	0.584	0.005	1.343	-0.240
**BD**	-1.336	0.563	0.826	0.046	-1.339	-0.203	1.179
**SCZ**	**0.113**	**1.668**	**0.868**	**-0.055**	**-0.112**	**0.239**	**-1.600**
**BMI**	-1.289	0.164	-0.160	0.071	1.871	1.096	-0.032
**Height**	-0.992	0.176	-0.097	0.073	2.305	0.018	0.083
**TG**	-0.604	0.851	0.655	0.244	2.692	0.431	0.529
**CD**	-0.433^*^	0.260	0.102	0.129	2.474	1.213	-0.110

* nominally significant

** significant after correction for multiple testing

Phenotypes: psychiatric and neurological diseases (Alzheimer’s disease (AD), bipolar disorder (BD), schizophrenia (SCZ)), anthropometric measures (body mass index (BMI), height), cardiovascular risk factors (Triglycerides (TG)), and immune-mediated diseases (Crohn’s disease (CD)). A: regression on the complete set of SNPs, B: regression analysis after removing MHC SNPs and C: regression analysis keeping only MHC SNPs. The enrichment seen in schizophrenia (**Fig 1B**) is not supported by regression where the association is significant (p = 6.00x10^-6^) but the effect is negative. The association loses significance upon removal of MHC. No significant associations are found when regressing MHC SNPs only.

**Table 3:** Ohnologs (Ohno): Partial least square regression (PLSR) coefficients with multiple covariates.

**A**							
**Phenotypes**	**Intron**	**Exon**	**3UTR**	**5UTR**	**Ohno**	**Brain**	**TotLD**
**AD**	2.126	0.664	0.846	0.192	-0.034	0.007	2.406
**BD**	0.437	0.678	0.534	0.314	3.001^**^	1.289	2.016^*^
**SCZ**	**0.214**	**1.677**	**2.984**	**1.277**	**0.496**	**0.383**	**0.443**
**BMI**	2.181	-0.258	0.100	0.056	0.477	1.104^*^	2.119
**Height**	2.443^**^	0.439	0.663	0.321	-0.424	0.553^**^	-0.645
**TG**	0.968	1.347	2.297	0.621	1.042	0.274	-0.950
**CD**	0.902	2.772	2.454	1.182	-0.039	0.661^*^	1.713^*^
**B**							
**Phenotypes**	**Intron**	**Exon**	**3UTR**	**5UTR**	**Ohno**	**Brain**	**TotLD**
**AD**	2.112	0.701	0.883	0.179	-0.027	2.423	0.010
**BD**	0.468	0.229	0.129	0.138	2.824^**^	1.595	0.962^*^
**SCZ**	**1.775**	**-0.915**	**2.246**	**0.577**	**0.757**	**0.953**	**0.873**
**BMI**	2.214	-0.256	0.112	0.055	0.399	2.068	1.098
**Height**	2.484^**^	0.225	0.491	0.206	-0.407	-0.563	0.467^**^
**TG**	0.970	1.068	2.427	0.562	1.255	-0.809	0.169
**CD**	1.310	2.816	2.674	1.221	0.122	2.008^*^	0.702^*^
**C**							
**Phenotypes**	**Intron**	**Exon**	**3UTR**	**5UTR**	**Ohno**	**Brain**	**TotLD**
**AD**	2.457	-0.248^*^	-0.168	0.196	1.194	0.629	-0.265
**BD**	-0.718	0.960	1.493	0.062	2.074	-0.328	0.463
**SCZ**	**-0.288**	**1.406**	**0.721**	**0.027**	0.620^**^	**-1.735**	**0.499**
**BMI**	-0.614	0.315^*^	-0.355	0.134^*^	-0.048	2.428	0.044
**Height**	-1.152	0.974	-0.659	0.386	1.862^*^	0.426	0.551
**TG**	-0.289	2.496	1.882	0.700	1.918	1.358	0.277
**CD**	-0.166	0.388^*^	0.127	0.186	-1.538	1.878	0.044

* nominally significant

** significant after correction for multiple testing

Phenotypes: psychiatric and neurological diseases (Alzheimer’s disease (AD), bipolar disorder (BD), schizophrenia (SCZ)), anthropometric measures (body mass index (BMI), height), cardiovascular risk factors (Triglycerides (TG)), and immune-mediated diseases (Crohn’s disease (CD)). A: regression on the complete set of SNPs, B: regression analysis after removing MHC SNPs and C: regression analysis keeping only MHC SNPs. The enrichment seen in schizophrenia (**Fig 1C**) is not supported by regression where no significant (p-value = 0.694) association is detected. The results remain non-significant after removing MHC SNPs but become significant when using only them.

The regression results are reflected by the squared z-scores versus TotLD scatter plots (**S2 Fig**). These show the small but significant effect of TotLD alongside that of stratification. HAR has no apparent effect on the squared z-scores regression while SD and Ohno seem to dampen the effect of TotLD, suggesting a possible interaction effect.

Removal of MHC has no relevant effects on PLSR results apart from a loss of significance of the SD association (**Tables 1–3B**). In light of these results, we repeated the regression analyses using only SNPs in the MHC region (**Tables 1–3C**). We observe that the regression coefficient for HAR changes direction but remains non-significant. In case of SD it remains negative but loses significance. The Ohno affiliation coefficient turns significant suggesting that Ohno affiliation might be an enrichment factor specific to the MHC region.

To determine the effect of LD on the enrichment analyses, we generated random sets of SNPs matching the original HAR, SD and Ohno SNPs in total LD and minor allele frequency and repeated the analyses on these sets. The enrichment is somewhat reduced (**S3 Fig**) compared to the original set of SNPs but is still present despite the lack of evolutionary content.

The specificity of the schizophrenia results was tested by repeating the same analyses for GWAS summary statistics of other human phenotypes: neurological and psychiatric (Alzheimer’s disease and bipolar disorder), anthropometric (body mass index (BMI), height), cardiovascular (triglycerides (TG)), immune-mediated (Crohn’s disease (CD)). These GWAS were selected to be roughly comparable in sample size to the schizophrenia GWAS and therefore roughly equally powered. The fold enrichment plots show an overall high enrichment in HAR and lower in SD and Ohno across all phenotypes (**S4 Fig**). The PLSR shows no significant associations in HAR, negative associations between SD affiliation and BD (non-significant) and BMI (nominally significant), but positive association between Ohno and BD (significant). Other covariates like intron affiliation score are positively associated, and significantly so, across most phenotypes when regressed together with HAR or SD scores.

## Discussion

We used polygenic enrichment methods to investigate whether SNPs in the HAR, SD and Ohno regions of the human genome, related to early evolution are more likely to be associated with schizophrenia. Our results did not provide clear evidence that genetic variants in these regions are more likely to associate with schizophrenia. While association enrichment is present, it appears to be mediated by affiliation to known genomic enrichment categories like introns, 3’UTR, 5’UTR, and LD. Thus, the risk variants associated with schizophrenia do not seem to have an evolutionary origin prior to divergence between humans and chimpanzees.

While association enrichment is present, it is likely mediated by affiliation to known genomic enrichment categories like introns, 3’UTR, 5’UTR, and LD.

HARs have the most recent origin among the three evolutionary proxies used in the current study [14]. HAR fold enrichment plots show some enrichment in schizophrenia, bipolar disorder and BMI, and more noteworthy enrichment in height, triglycerides and Crohn’s disease. However, regression analyses yielded no significant results in any phenotype, likely owing to the small number of SNPs in these regions. SD and Ohno show similar patterns of enrichment but generally less pronounced than in HAR. This suggests that the enrichment is not specific for schizophrenia but is influenced by the age and the extent of the evolutionary markers. We do observe significant and positive correlation between Ohno and BD but this is likely spurious since none of the GWAS from larger samples, including the schizophrenia GWAS, show any significant results.

Removal of MHC had the most visible effects on HAR followed by SD enrichments. HAR and SD, likely owing to their more recent origin, contain a higher proportion of MHC genes compared to Ohno (**S2 and S3 Tables**). Ohnos are the oldest regions and cover large portions of the human genome [25]. As such, while still confounded by LD, their enrichment is largely determined by SNPs with low LD scores and hence less likely to tag any causal variants. The removal of MHC had no visible effect on Ohno enrichment but our analysis of SNPs in MHC regions interestingly yielded a significant positive association, suggesting the presence of some evolutionary effect specific to the MHC [44].

Since brain genes are more likely to be associated with schizophrenia, we carried out separate analyses targeting these. Our results suggest that brain SNPs in evolutionary salient regions show enrichment of associations with schizophrenia compared to just any brain genes or non-brain genes within the same regions. Interestingly, regions that are shared by Ohno and SD show greater enrichment of associations with schizophrenia compared to either of them separately. However, this could be due to higher LD in these shared regions. SNPs with higher LD scores are likely to tag both Ohno and SD regions as much as they are likely to be associated with any causal variants.

Our analyses on random sets of SNPs matching the original ones suggest that most of the enrichment in the evolutionary salient regions is due to LD. However, a residual enrichment is clearly visible in SD and Ohno and can be guessed in HAR despite its larger variance (**S3 Fig**). Judging by the PLSR results, such residual enrichment should probably be attributed to Introns and UTRs **(Tables 1–3).** This is likely due to overall higher gene content in the case of SD and Ohno (**S3 Table**). The culprit in the case of HAR is likely to be found in LD with non-coding regulatory elements.

It has been proposed that schizophrenia is the price we paid for the development of a complex brain and our ability to have abstract reasoning and language [45]. The schizophrenia association enrichment in evolutionary markers appears to decrease as the age of the evolutionary markers increases. However, the extent of the LD-weighted evolutionary markers studied here must also be considered. Limited enrichment sensitivity may also be a reason for the lack of findings in the present study, especially in the case of HAR. Other investigators found HAR to be enriched in schizophrenia [46]. However, they distinguished HAR that have accelerated in humans compared to non-human primates (pHAR) and compared to other mammals (mHAR). Further, their analyses were restricted to schizophrenia GWAS loci below given significance levels. They used LD (r2 > 0.5) to define their regions of interest while we treat LD as a confounder and control for it. These factors may be the reason for our failure to reproduce their findings. However, our polygenic techniques were able to show enrichment in regions under positive selection in humans after divergence from Neanderthals [9].

The modern human genome is the result of a complex process of evolution and adaptation. Using multiple evolutionary markers we investigated traces of human-specific pathology in the ancestral regions of the human genome. Our investigation did not reveal conclusive evidence supporting increased prevalence of schizophrenia association in the markers of early human evolution, HAR, SD or Ohno. Taken together with our previous report on markers of human-Neanderthal divergence [9], the current findings suggest that the increase in polygenic schizophrenia risk may have developed during more recent periods of human evolution.

## Supporting Information

S1 Table A: GWASs with available summary statistics. List of genome wide association studies (GWAS) analyzed. The table shows the phenotypes, the sample size (i.e. the number of subjects, N); the total number of SNPs entering our analyses. PGC: Psychiatric Genomics Consortium, results from the second edition of the study.
(DOCX)

S2 Table Distribution of evolutionarily salient SNPs in each genomic category. The table shows the number of SNPs that are affiliated to human accelerated regions (HAR), segmental duplications (SD), ohnologs (Ohno) and their distribution in various genomic categories.
(DOCX)

S3 Table Odds Ratio. Table of odds ratios of HAR, SD and Ohno SNPs affiliation in each genomic category.
(DOCX)

S1 Fig Enrichment plots for schizophrenia, stratifying SNPs according to their affiliation to human accelerated regions segmental duplications and ohnologs after removing MHC SNPs. Plot **A** shows all SNPs stratified by affiliation to human accelerated regions (HAR) and non (NonHAR). Plot **B** shows all SNPs stratified by affiliation to segmental duplications (SDLD) and non (NonSDLD). Plot **C** shows all SNPs stratified by affiliation to ohnologous (OhnoLD) and non ohnologous regions (NonOhno). We see some visible depletion of the enrichment in SD but none in HAR or ohnologs.
(TIF)

S2 Fig Stratified squared z-scores vs Total LD plots for schizophrenia. 1A: human accelerated regions (HAR), 1B: Segmental duplications (SD), 1C: Ohnologs (Ohno). The SNPs are stratified based on their regional affiliation score: HAR, SD, Ohno vs non HAR, non SD, non Ohno, respectively. A scatter plot for all SNPs (None) is also reported in all figures. Mean squared z-scores are lower in SD and Ohno compared to non SD and non Ohno (Fig 1B and 1C). HAR has no apparent effect on squared z-scores. Non HAR, non SD and non Ohno essentially overlap with the non-stratified set (None).
(TIF)

S3 Fig Enrichment plots for schizophrenia, conditioning SNPs according to their affiliation to SNP sets matched to ohnologs, segmental duplications and human accelerated regions in minor allele frequency and total LD. **Plot A** shows all SNPs stratified by affiliation to human accelerated regions (HAR) and non HAR. **Plot B** shows all SNPs stratified by affiliation to segmental duplication (SDLD) and non-segmental duplication regions (NonSD); **Plot C** shows all SNPs in ohnologous (Ohno) and non ohnologous regions (NonOhno). We observe some depletion of enrichment compared to the original set of SNPs but it is still present despite the lack of evolutionary content.
(TIF)

S4 Fig Enrichment plots for some representative phenotypes, conditioning SNPs by their affiliation to human accelerated regions, segmental duplications and ohnologs. The phenotypes are Alzheimer’s disease (AD), bipolar disorder (BD), body mass index (BMI), height, triglycerides (TG) and Crohn’s disease (CD). **Plot A** shows all SNPs stratified by affiliation to human accelerated regions (HAR) and non HAR; **Plot B** shows all SNPs stratified by affiliation to segmental duplication (SDLD) and non-segmental duplication regions (NonSD); **Plot C** shows all SNPs in ohnologous (Ohno) and non ohnologous regions (NonOhno). We observe some enrichment for HAR and more clear enrichment for segmental duplications but a weak enrichment for ohnologs.
(TIF)

S5 Fig Enrichment plots for some representative phenotypes, conditioning SNPs by their affiliation to LD informed annotations based on human accelerated regions, segmental duplication and ohnologs after removing MHC SNPs. The phenotypes are Alzheimer’s disease (AD), bipolar disorder (BD), body mass index (BMI), Height, triglycerides (TG) and crohn’s disease (CD) **Plot A** shows SNPs stratified by affiliation to human accelerated regions (HAR) and non HAR. **Plot B** shows SNPs stratified by affiliation to segmental duplication (SD) and non-segmental duplication regions (nonSD); **Plot C** shows SNPs in ohnologous (Ohno) and non ohnologous regions (NonOhno). We observe some depletion of enrichment in HAR and segmental duplications but none for ohnologs.
(TIF)

S1 File Author notes. (DOCX)
