# Supplementary material for: Probing the Association between Early Evolutionary Markers and Schizophrenia
Source: PLoS One. 2017 Jan 12;12(1):e0169227. doi: 10.1371/journal.pone.0169227 (PMC5231388; doi:10.1371/journal.pone.0169227)
Supplement: S1 Table — List of genome wide association studies (GWAS) analyzed. The table shows the phenotypes, the sample size (i.e. the number of subjects, N); the total number of SNPs entering our analyses. PGC: Psychiatric Genomics Consortium, results from the second edition of the study. (DOCX) [file pone.0169227.s001.docx]

**S1 Table. GWASs with available summary statistics.**

| **Phenotype** | | **Sample size (N)** | **Number of SNPs** |
| --- | --- | --- | --- |
|  |  |  | **Total** |
| CNS/ Neurological Disorders | Alzheimer’s Disease (1) | 54,162 | 2,436,961 |
|  |  |  |  |
| Psychiatric disorders | Bipolar disorder (2) | 16,731 | 2,540,792 |
|  | Schizophrenia (3) | 82,315 | 2,540,803 |
| Anthropometric Measures | Body mass index (4) | 339,224 | 2,433,922 |
|  |  |  |  |
|  | Height (5) | 183,727 | 2,398,527 |
| Cardiovascular disease | Triglycerides (6) | 96,568 | 2,508,363 |
| (CVD) risk factors |  |  |  |
| Immune-mediated disease | Crohn's disease (7) | 51,109 | 942,858 |

List of genome wide association studies (GWAS) analyzed. The table shows the phenotypes, the sample size (i.e. the number of subjects, N); the total number of SNPs entering our analyses. PGC: Psychiatric Genomics Consortium, results from the second edition of the study.

#### References

1. Lambert JC, Ibrahim-Verbaas CA, Harold D, Naj AC, Sims R, Bellenguez C, et al. (2013): Meta-analysis of 74,046 individuals identifies 11 new susceptibility loci for Alzheimer's disease. *Nature genetics*. 45:1452-1458.

2. Psychiatric GCBDWG (2011): Large-scale genome-wide association analysis of bipolar disorder identifies a new susceptibility locus near ODZ4. *Nature genetics*. 43:977-983.

3. Schizophrenia Working Group of the Psychiatric Genomics C (2014): Biological insights from 108 schizophrenia-associated genetic loci. *Nature*. 511:421-427.

4. Locke AE, Kahali B, Berndt SI, Justice AE, Pers TH, Day FR, et al. (2015): Genetic studies of body mass index yield new insights for obesity biology. *Nature*. 518:197-206.

5. Lango Allen H, Estrada K, Lettre G, Berndt SI, Weedon MN, Rivadeneira F, et al. (2010): Hundreds of variants clustered in genomic loci and biological pathways affect human height. *Nature*. 467:832-838.

6. Teslovich TM, Musunuru K, Smith AV, Edmondson AC, Stylianou IM, Koseki M, et al. (2010): Biological, clinical and population relevance of 95 loci for blood lipids. *Nature*. 466:707-713.

7. Franke A, McGovern DP, Barrett JC, Wang K, Radford-Smith GL, Ahmad T, et al. (2010): Genome-wide meta-analysis increases to 71 the number of confirmed Crohn's disease susceptibility loci. *Nature genetics*. 42:1118-1125.
