# Supplementary material for: Probing the Association between Early Evolutionary Markers and Schizophrenia
Source: PLoS One. 2017 Jan 12;12(1):e0169227. doi: 10.1371/journal.pone.0169227 (PMC5231388; doi:10.1371/journal.pone.0169227)
Supplement: S2 Table — The table shows the number of SNPs that are affiliated to human accelerated regions (HAR), segmental duplications (SD), ohnologs (Ohno) and their distribution in various genomic categories. (DOCX) [file pone.0169227.s002.docx]

##### S2 Table: Distribution of evolutionarily salient SNPs in each genomic category

| **Covariate** | **Intron** | **Exon** | **3UTR** | **5UTR** | **MHC** | **Total** |
| --- | --- | --- | --- | --- | --- | --- |
| HAR | 1,159 | 261 | 168 | 126 | 117 | 2878 |
| SD | 231,308 | 100,602 | 99,523 | 38,548 | 6,911 | 308,537 |
| Ohno | 1,049,828 | 226,832 | 248,443 | 76,275 | 8,220 | 1,123,588 |

The table shows the number of SNPs that are affiliated to human accelerated regions (HAR), segmental duplications (SD), ohnologs (Ohno) and their distribution in various genomic categories.
