# Supplementary material for: Probing the Association between Early Evolutionary Markers and Schizophrenia
Source: PLoS One. 2017 Jan 12;12(1):e0169227. doi: 10.1371/journal.pone.0169227 (PMC5231388; doi:10.1371/journal.pone.0169227)
Supplement: S3 Table — Table of odds ratios of HAR, SD and Ohno SNPs affiliation in each genomic category. (DOCX) [file pone.0169227.s003.docx]

**S3 Table: Odds Ratio**

| **Covariate** | **Intron** | **Exon** | **3UTR** | **5UTR** | **MHC** |
| --- | --- | --- | --- | --- | --- |
| HAR | 0.48^*^ | 0.70^*^ | 0.42^*^ | 1.14 | 8.41^*^ |
| SD | 2.33^*^ | 4.54^*^ | 4.15^*^ | 5.22^*^ | 8.54^*^ |
| Ohno | 31.52^*^ | 3.77^*^ | 4.63^*^ | 4.63^*^ | 2.24^*^ |

* significant after accounting for multiple testing

Table of odds ratios of HAR, SD and Ohno SNPs affiliation in each genomic category.
