# Supplementary figures and images for: Probing the Association between Early Evolutionary Markers and Schizophrenia
Source: PLoS One. 2017 Jan 12;12(1):e0169227. doi: 10.1371/journal.pone.0169227 (PMC5231388; doi:10.1371/journal.pone.0169227)

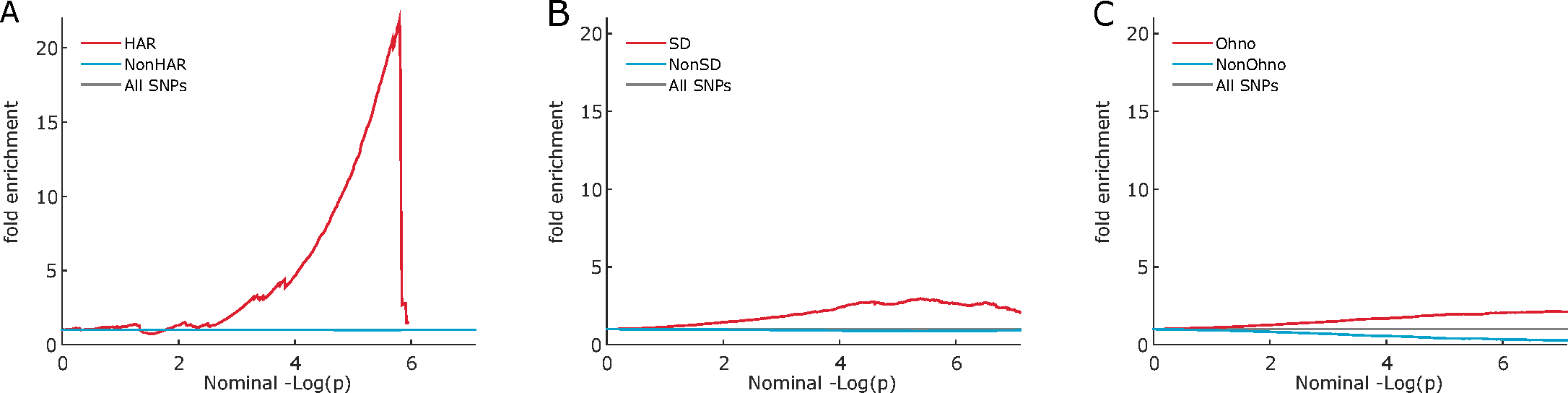

Supplement: S1 Fig — Plot A shows all SNPs stratified by affiliation to human accelerated regions (HAR) and non (NonHAR). Plot B shows all SNPs stratified by affiliation to segmental duplications (SDLD) and non (NonSDLD). Plot C shows all SNPs stratified by affiliation to ohnologous (OhnoLD) and non ohnologous regions (NonOhno). We see some visible depletion of the enrichment in SD but none in HAR or ohnologs. (TIF) [file pone.0169227.s004.tif]

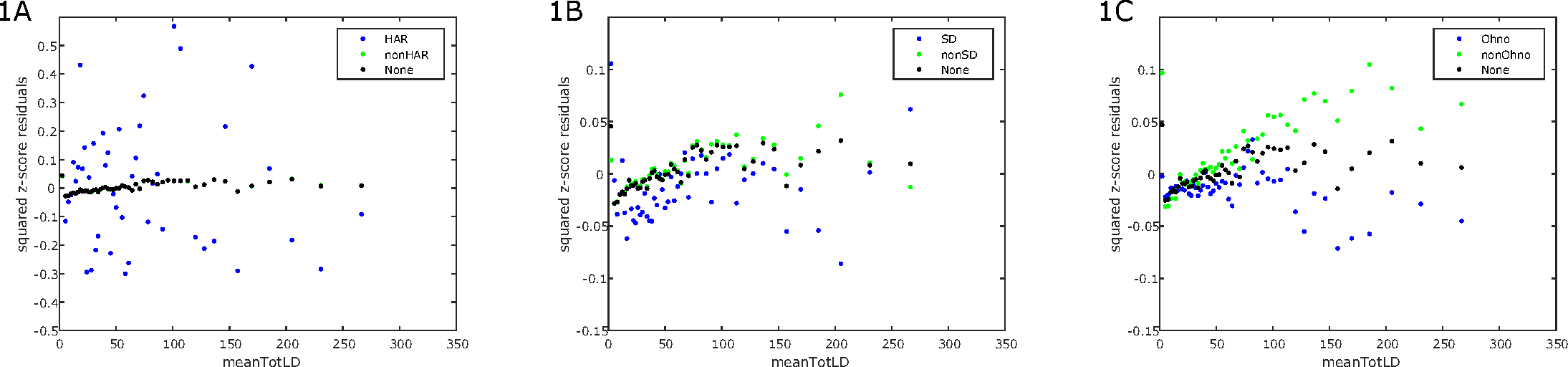

Supplement: S2 Fig — 1A: human accelerated regions (HAR), 1B: Segmental duplications (SD), 1C: Ohnologs (Ohno). The SNPs are stratified based on their regional affiliation score: HAR, SD, Ohno vs non HAR, non SD, non Ohno, respectively. A scatter plot for all SNPs (None) is also reported in all figures. Mean squared z-scores are lower in SD and Ohno compared to non SD and non Ohno (Fig 1B and 1C). HAR has no apparent effect on squared z-scores. Non HAR, non SD and non Ohno essentially overlap with the non-stratified set (None). (TIF) [file pone.0169227.s005.tif]

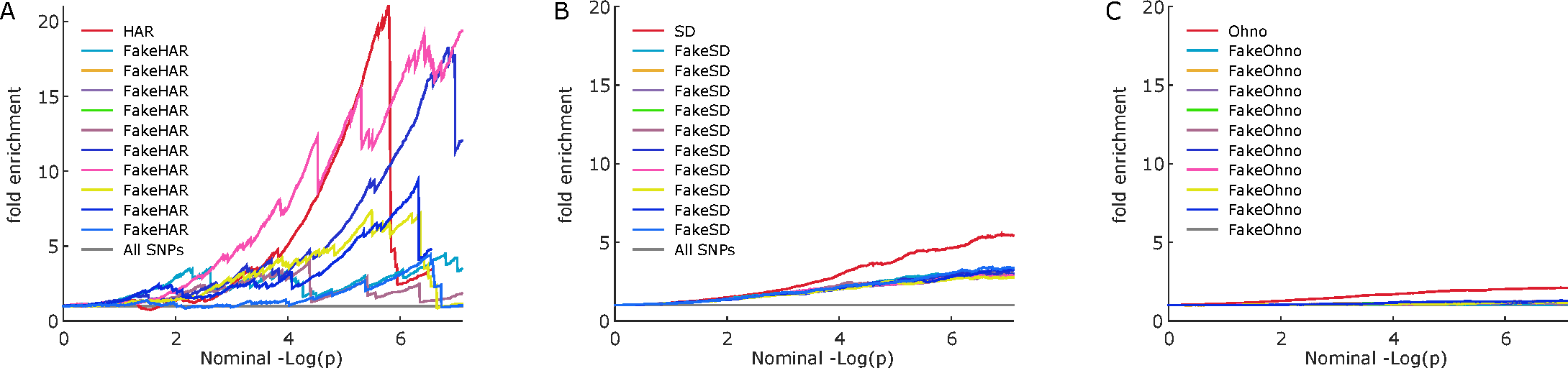

Supplement: S3 Fig — Plot A shows all SNPs stratified by affiliation to human accelerated regions (HAR) and non HAR. Plot B shows all SNPs stratified by affiliation to segmental duplication (SDLD) and non-segmental duplication regions (NonSD); Plot C shows all SNPs in ohnologous (Ohno) and non ohnologous regions (NonOhno). We observe some depletion of enrichment compared to the original set of SNPs but it is still present despite the lack of evolutionary content. (TIF) [file pone.0169227.s006.tif]

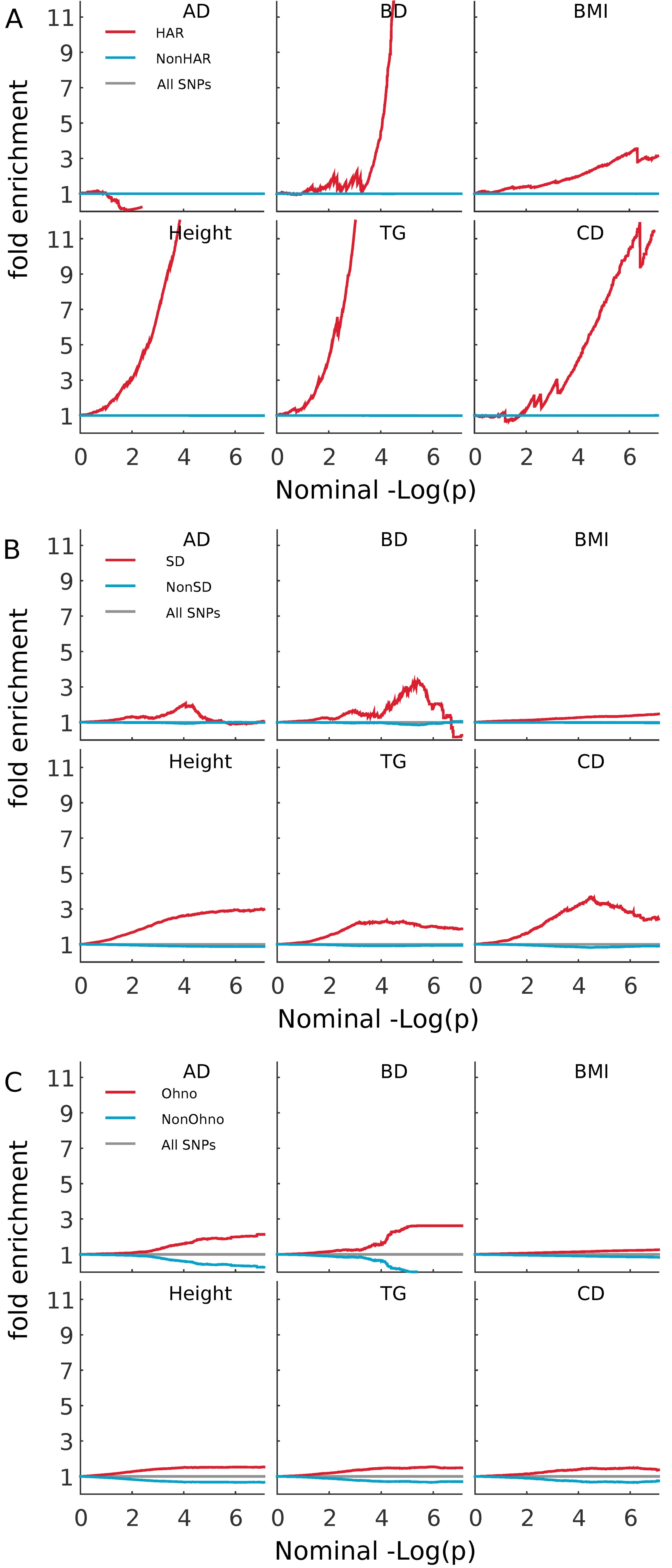

Supplement: S4 Fig — The phenotypes are Alzheimer’s disease (AD), bipolar disorder (BD), body mass index (BMI), height, triglycerides (TG) and Crohn’s disease (CD). Plot A shows all SNPs stratified by affiliation to human accelerated regions (HAR) and non HAR; Plot B shows all SNPs stratified by affiliation to segmental duplication (SDLD) and non-segmental duplication regions (NonSD); Plot C shows all SNPs in ohnologous (Ohno) and non ohnologous regions (NonOhno). We observe some enrichment for HAR and more clear enrichment for segmental duplications but a weak enrichment for ohnologs. (TIF) [file pone.0169227.s007.tif]

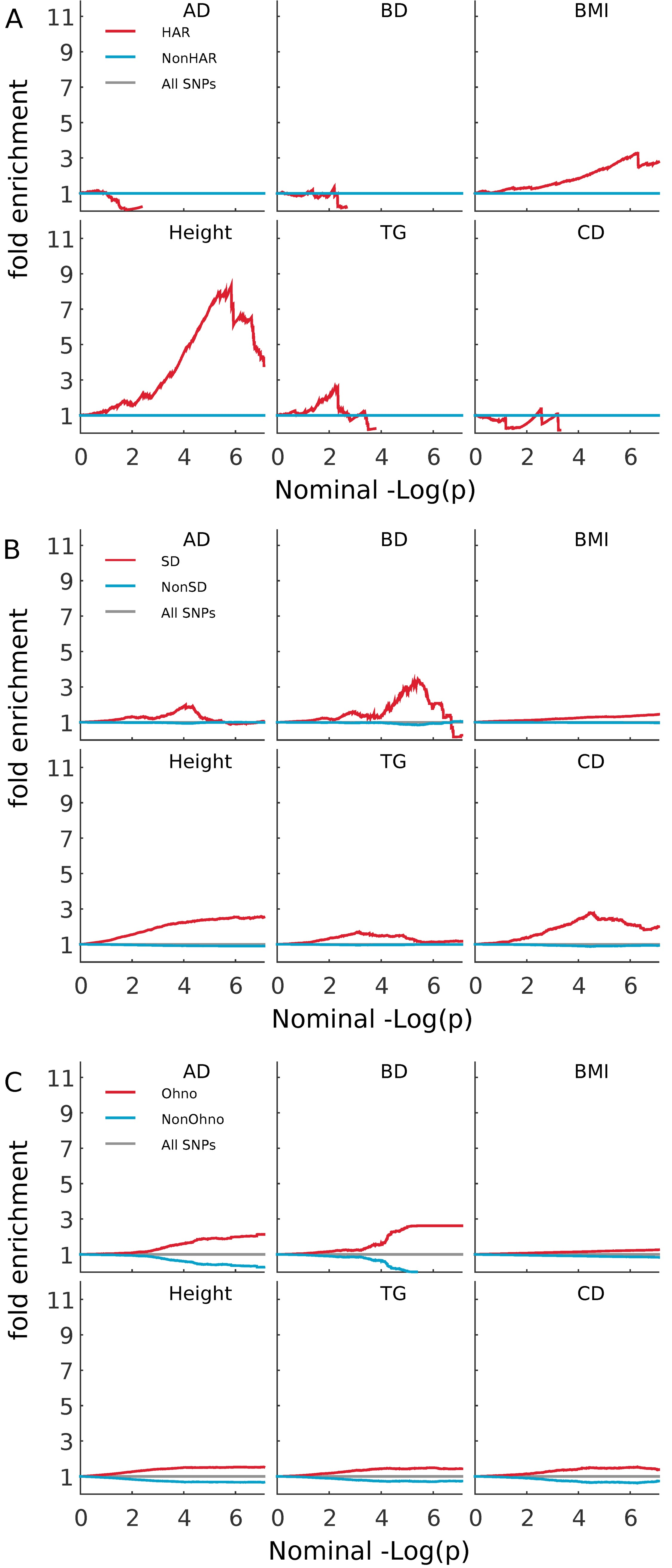

Supplement: S5 Fig — The phenotypes are Alzheimer’s disease (AD), bipolar disorder (BD), body mass index (BMI), Height, triglycerides (TG) and crohn’s disease (CD) Plot A shows SNPs stratified by affiliation to human accelerated regions (HAR) and non HAR. Plot B shows SNPs stratified by affiliation to segmental duplication (SD) and non-segmental duplication regions (nonSD); Plot C shows SNPs in ohnologous (Ohno) and non ohnologous regions (NonOhno). We observe some depletion of enrichment in HAR and segmental duplications but none for ohnologs. (TIF) [file pone.0169227.s008.tif]
