# Supplementary material for: Probing the Association between Early Evolutionary Markers and Schizophrenia
Source: PLoS One. 2017 Jan 12;12(1):e0169227. doi: 10.1371/journal.pone.0169227 (PMC5231388; doi:10.1371/journal.pone.0169227)
Supplement: S1 File — (DOCX) [file pone.0169227.s009.docx]

### Author Notes

We thank the International Genomics of Alzheimer's Project (IGAP) for providing summary results data for these analyses. The investigators within IGAP contributed to the design and implementation of IGAP and/or provided data but did not participate in analysis or writing of this report. IGAP was made possible by the generous participation of the control subjects, the patients, and their families. The i–Select chip was funded by the French National Foundation on Alzheimer's disease and related disorders. EADI was supported by the LABEX (laboratory of excellence program investment for the future) DISTALZ grant, Inserm, Institut Pasteur de Lille, Université de Lille 2 and the Lille University Hospital. GERAD was supported by the Medical Research Council (Grant n° 503480), Alzheimer's Research UK (Grant n° 503176), the Wellcome Trust (Grant n° 082604/2/07/Z) and German Federal Ministry of Education and Research (BMBF): Competence Network Dementia (CND) grant n° 01GI0102, 01GI0711, 01GI0420. CHARGE was partly supported by the NIH/NIA grant R01 AG033193 and the NIA AG081220 and AGES contract N01–AG–12100, the NHLBI grant R01 HL105756, the Icelandic Heart Association, and the Erasmus Medical Center and Erasmus University. ADGC was supported by the NIH/NIA grants: U01 AG032984, U24 AG021886, U01 AG016976, and the Alzheimer's Association grant ADGC–10–196728.

YW has received funding from The Research Council of Norway through a FRIPRO Mobility Grant, contract no 251134. The FRIPRO Mobility grant scheme (FRICON) is co-funded by the European Union’s Seventh Framework Programme for research; technological development and demonstration under Marie Curie grant agreement no 608695. Eli Lilly & Co. UK provided support in the form of salary for the author [DAC], but did not have any additional role in the study design, data collection and analysis, decision to publish, or preparation of the manuscript and has no commercial interests in this study. The company does not alter the authors’ adherence to PLOS policy on data sharing. The affiliation of DAC to Eli Lilly & Co. has no effect on the study and any relation to patents, products in development, or marketed products.
